# Nasal microbiota profiles are similar at two swabbing depths in healthy awake dogs

**DOI:** 10.3389/fvets.2026.1795324

**Published:** 2026-04-28

**Authors:** Attila Salamon, Soufiane Bel Rhali, Attila Szabó, Ádám Miklósi, Attila Andics, Tamás Felföldi, Márta Gácsi, Enikő Kubinyi

**Affiliations:** 1 Department of Ethology, ELTE Eötvös Loránd University, Budapest, Hungary; 2 HUN-REN-ELTE Comparative Ethology Research Group, Budapest, Hungary; 3 NAP Canine Brain Research Group, ELTE Eötvös Loránd University, Budapest, Hungary; 4 Department of Microbiology, ELTE Eötvös Loránd University, Budapest, Hungary; 5 Doctoral School of Biology, Institute of Biology, ELTE Eötvös Loránd University, Budapest, Hungary; 6 Department of Aquatic Sciences and Assessment, Swedish University of Agricultural Sciences, Uppsala, Sweden; 7 Neuroethology of Communication Lab, Department of Ethology, Eötvös Loránd University, Budapest, Hungary; 8 MTA-ELTE Lendület “Momentum” Companion Animal Research Group, Budapest, Hungary

**Keywords:** animal welfare, diversity, dog, *Moraxella*, nasal microbiota, sampling method

## Abstract

The nasal microbiome may play an important role in dogs’ olfaction, as microbial communities in the nasal cavity can directly interact with the olfactory epithelium. Previous studies have used various sampling methods and depths to examine the canine nasal microbiota and the effect of sampling depth on the detected nasal microbiota remains unclear. To address this, we investigated and compared the nasal microbiota of 81 awake family dogs, sampled at two swabbing depths (15 mm and 25 mm) of the left nostril in the same individuals. The most abundant bacterial taxa in our nasal samples belonged to *Moraxella* (*Gammaproteobacteria*), consistent with previous studies. The 15 mm samples had a higher Shannon Diversity Index compared to the 25 mm samples, indicating closer proximity to the external environment. However, we found no significant differences in richness and overall microbiota composition between the two sampling depths. These results indicate that bacterial community profiles within the anterior nasal cavity are similar at the two examined depths. Because the deeper sample was always collected first from the same nostril, potential order effects cannot be excluded. Nevertheless, based on the findings, we recommend using a sampling depth up to 15 mm when examining the nasal microbiota of healthy awake mesocephalic dogs in field settings, as it is less invasive and minimises discomfort.

## Introduction

Nasal microbiota in mice plays a crucial role in maintaining nasal health and may potentially influence olfactory function (1, 2). Based on these findings that microbial communities in the nasal cavity may interact directly or indirectly with the olfactory epithelium, it is suggested that variations in the nasal microbiota contribute to differences in dogs’ olfactory performance (3), as has been shown in humans (4). However, to investigate this connection, it is first necessary to develop a standardized method for collecting samples from the nasal cavity of animals. Identifying sampling approaches that are minimally invasive yet yield comparable microbiota profiles would improve the consistency of canine nasal microbiota studies. Such a technique will ultimately allow meaningful comparisons between individuals and across different conditions.

The few studies describing dogs’ nasal microbiota (5–8) vary in sampling methods (Table 1), but all found that the most common nasal bacteria in dogs belong to the phylum *Proteobacteria* (recently renamed as *Pseudomonadota*), the family *Moraxellaceae* and the genus *Moraxella* [reviewed in Pereira and Clemente (9); see Table 1]. These studies confirmed that both extrinsic and intrinsic factors affect the composition of the upper airway microbiota in dogs. These include spatial variation between nasal and oropharyngeal swabs (5, 7), temporal variation between two sampling occasions 7 weeks apart (7), changes with age and body weight (6), head shape (10), as well as with the geographical location of the subjects (7).

**Table 1:** Previous studies that examined dogs’ nasal microbiota communities.

Study	N/sex/breed/age	Sampling method, hypervariable regions examined, microbial community profiling	Taxon level	Most common taxa (> 10% mean relative abundance)
Ericsson et al. (5)	16, all females lab beagles, 24–96 months	Moistened swab inserted midway between the tip of the nose and the medial canthus of the eye, and rotated vigorously during anesthesia, V4, OTU	Phyla	*Proteobacteria* (55.4%) *Tenericutes* (28.0%)
			Families	*Moraxellaceae* (33.0%) *Mycoplasmataceae* (28.0%)
			Genera	*Moraxella* (20.5%) *Mycoplasma* (28.0%)
Tress et al. (6)	23, females (*N* = 14) males (*N* = 9), 10 breeds and 11 mongrels, 10–132 months	Sterile dry rayon swab inserted into each nostril and rotated carefully while awake, V4, OTU	Phylum	*Proteobacteria* (82.8%)
			Family	*Moraxellaceae* (60.5%)
			Genus	*Moraxella* (58.0%)
Isaiah et al. (7)	Exp1: 81, females (*N* = 44) males (*N* = 37), 11 breeds and 16 mongrels, 1–12 years	Sterile swabs inserted approximately half an inch into the nasal cavity and swabbed, V4-V6, OTU	Phyla	*Proteobacteria* (42.3%) *Bacteroidetes* (20.8%)
			Family	*Moraxellaceae* (35.6%)
			Genus	*Moraxella* (35.0%)
Vangrinsven et al. (8)	29, females (*N* = 17) males (*N* = 12), 11 breeds and 1 mongrel, 0.8–11.3 years	A sterile speculum inserted into one nare to allow the passage of a sterile swab in the distal third of the nasal cavity during anesthesia, V2-V3, OTU	Phyla	*Proteobacteria* (71.6%) *Firmicutes* (10.3%)
			Family	*Moraxellaceae* (52.0%)
			Fenus	*Moraxella* (51.8%)

In the study of Isaiah et al. (7), medians were reported instead of means. Note that phylum Proteobacteria was recently renamed as Pseudomonadota, Tenericutes as Mycoplasmatota, and Bacteriodetes as Bacteroidota and Firmicutes as Bacillota.

There is a notable gap in the literature concerning the comparison of the effectiveness of various nasal sample collection methods. It has been confirmed in humans that swabbing can yield as good a sample as a mucosal tissue sample for examining the nasal microbiota (11), although extrapolation of such results to dogs is limited due to the considerable anatomical and functional differences between them. However, no standardized swabbing method currently exists for dogs. All studies sampled a different depth of the nasal cavity, which is difficult to compare, as only one study gave an exact number (half an inch: 7), while the others gave approximations based on the dogs’ facial/anatomical features (midway between the tip of the nose and the medial canthus of the eye: 5; distal third of the nasal cavity: 10) or not reported the sampling depth (6). This is further complicated by the fact that dogs have a high variability in head shape and size (12). Another issue is that most studies conducted sampling under anaesthesia (healthy dogs: 5, 8, 10; dogs with nasal disease: 6, 8). Anaesthesia is often required for proper sampling of the deeper nasal subsites, but nasal mucosal swabbing in the nasal cavity can be performed with minimal discomfort (13). While anaesthesia can improve sampling precision by reducing the risk of swab contamination from the nares and upper airways (10), it is an adjunct procedure, which may pose some risk to the animal. Therefore, performing anaesthesia on healthy dogs raises ethical concerns, especially when alternative methods are available. Additionally, it requires a veterinarian and specialized equipment, making it impractical for large-scale or field studies. Furthermore, since the major bacterial taxa identified were consistent across previous studies, contamination from touching the nares during sampling may not be a significant concern. In awake dogs, completely avoiding contact with the nostrils is nearly impossible, making it a practical limitation and a critical flaw in sample collection.

In the present exploratory study, we aimed (i) to examine the nasal microbiota of awake healthy family dogs and (ii) to compare samples collected at ~15 mm and 25 mm depths, which is still achievable with minimal discomfort without anesthesia. We expected no significant difference between the two sampling depths in richness, diversity or the overall nasal microbiota community composition in a large and diverse population of family dogs.

## Materials and methods

### Ethics statement

The data collection of the current study was connected to an olfactory test (14–16) for which ethical approvals were granted by the Animal Welfare Committee of Eötvös Loránd University (ELTE-AWC-020/2018 and ELTE-AWC-015/2023). All methods were carried out in accordance with relevant guidelines (including ARRIVE) and regulations. The experiment was performed in accordance with the EU Directive 2010/63/EU and the recommendations of the Hungarian State Health and Medical Service. Companion dog owners were recruited through social media and from the Family Dog Project database (Department of Ethology, Eötvös Loránd University). Informed consent was also obtained from all owners, and they participated in the test and data collection with their dogs on a voluntary basis. Owners could terminate the experiment and data collection at any time.

### Subjects

Samples were collected from 101 companion dogs living in Hungarian households in 2021–2022. From these, we excluded 10 dogs due to sampling and handling problems (see Sample collection) and 10 dogs due to low quality or insufficient sequencing data (see Data pre-processing and statistical analysis). Thus, we analyzed the data of 81 dogs from 11 mesocephalic breeds (2 Basset hounds, 7 beagles, 2 Belgian shepherd dog, 12 border collies, 7 cocker spaniels, 6 German pointers, 3 German shepherd dogs, 13 golden retrievers, 16 Labrador retrievers, 12 Hungarian vizslas, 1 Weimaraner), containing 36 males (23 neutered) and 45 females (32 neutered), with ages ranging from 0.5 to 16 years (mean: 5.6, SD: 3.8 years). All subjects were reported to be healthy by their owners, based on their current behaviour, appetite, and general well-being. None of them was under medication (e.g., antidepressants, antibiotics, probiotics) during or in the preceding month of the sampling.

### Sample collection

To describe the dog’s nasal microbiota, two nasal swabs were taken at two different depths immediately after the dogs had participated in an olfactory test (14–16); one at 25 mm and another at 15 mm depth. The length was measured with a ruler and marked on the swab’s stalk; the swab was inserted into the dog’s nose until the mark. The samples were taken from the same nostril because dogs preferentially use the right nostril to sniff conspecific arousal odors and novel odors, and the left nostril to sniff familiar odors and non-aversive stimuli, such as food (3), which could affect microbiota composition in each nostril. For this purpose, the sterile, dry swab (Copan®, FLOQSwabs™, 518C or 501CS01, Brescia, Italy; depending on availability) was inserted into the left nostril along the nasal septum and twisted gently around its axis. (The two types of swabs were functionally identical; the only difference was that the Copan® FLOQSwabs™ 518C came with its own tube, while the Copan® FLOQSwabs™ 501CS01 came without a tube.) The collection procedure was conducted while the dogs were awake, with the owners’ active help. These samples were preserved at −80 °C within a 15-min timeframe. The sampling was done in our behavioural lab, which is adjacent to the lab where we keep our deep freezers, so sample processing within this timeframe is possible. The behavioural lab was not an unfamiliar environment to the participating dogs, as they regularly visit for various behavioural tests. Further, the owner was present during the sampling, which would ease the stress. We always took the 25 mm sample first, because based on the pilot tests, the procedure might have caused discomfort for some dogs and swabbing a second time could inflict resistance against the procedure. The data from 10 dogs were excluded from the analysis as they had only one sample due to several reasons. Six of these samples, one sample per dog, were classified as 15 mm samples because the swabs could not be inserted deeper without causing clear signs of discomfort (e.g., head shaking, attempts to withdraw, vocalisation, licking or biting the swab). For four additional dogs, one sample each was lost during handling or processing.

### 16S rRNA amplicon sequencing

The nasal microbiota was analysed using partial amplicon sequencing of the bacterial 16S rRNA gene. Total genomic DNA extraction was performed using the DNeasy PowerSoil Pro kit following the instructions given by the manufacturer (protocol from 2019), except that the cell disruption was performed by shaking at 30 Hz for 2 min using a Retsch MM400 mixer mill. PCRs were performed with primers B341F [5′- CCT ACG GGN GGC WGC AG -3′; Herlemann et al. (17)] and 805NR [5′- GAC TAC NVG GGT ATC TAA TCC -3′; Apprill et al. (18)] targeting the V3-V4 region of the 16S rRNA gene as it was described in detail in Lange-Enyedi et al. (19). DNA sequencing was performed on an Illumina MiSeq platform using 2x250bp paired end format and MiSeq standard v2 chemistry, as a service provided by the Genomics Core Facility RTSF, Michigan State University, East Lansing MI, USA. Controls applied during the procedures performed in this study are given in Supplementary Table 1.

### Data pre-processing and statistical analysis

Bioinformatic analysis of raw sequence reads was carried out using Mothur v1.48 (20), as described in Salamon et al. (21). Eleven samples from 10 dogs were discarded from the dataset due to the low number of high-quality sequences. For statistical analyses, reads were subsampled to the read number of the sample having the lowest sequence count (*n* = 12,476). Raw sequence reads have been deposited in the NCBI Sequence Read Archive under BioProject ID PRJNA1371937.

Calculation of richness (observed OTUs, operational taxonomic units assigned at a 97% nucleotide sequence similarity level) and diversity (Shannon Diversity Index) estimators was also performed using Mothur. Non-metric multidimensional scaling (NMDS) was carried out in R [v4.4.2; R Development Core Team (22)] using the packages ‘vegan’ v2.6.10 (23) and ‘ggplot2’ v4.0.0 (24). The normality of richness and diversity data was assessed using the Shapiro–Wilk test, and differences between the two sampling depths were tested using the non-parametric Wilcoxon signed-rank test in R. For the determination of significant differences in community composition between outer and inner nasal samples, a two-way PERMANOVA test was applied based on Bray–Curtis similarity with 999 permutations using the ‘adonis2’ function of vegan. We also performed a similarity percentage (SIMPER) test using the PAST v3 software (25) to determine the OTUs responsible for the dissimilarity among the two sampling depths.

## Results

### Nasal microbiota composition

A total of 8,747,238 high-quality bacterial 16S rRNA gene sequences were obtained from 162 nasal samples collected from 81 dogs, corresponding to 53,995 ± 24,666 reads per sample.

Two major phyla (represented by > 10% mean relative abundance of the total bacterial community on average) were identified in the dogs’ nasal microbiota: *Proteobacteria* and *Firmicutes* (Table 2). Also, one major family, *Moraxellaceae* (> 10% mean relative abundance on average), and one major genus, *Moraxella* (> 10% mean relative abundance on average), were identified (Table 2).

**Table 2:** Mean relative abundance of microbial taxa identified in the nasal microbiota of 81 companion dogs from two depths.

Taxa	25 mm nasal depth		Taxa	15 mm nasal depth	
	Mean (%)	SD		Mean (%)	SD
Phyla					
*Proteobacteria*	67.53	22.73	*Proteobacteria*	63.45	22.88
*Firmicutes*	15.56	19.84	*Firmicutes*	18.39	19.60
*Actinobacteriota*	7.34	8.17	*Actinobacteriota*	6.72	6.00
*Patescibacteria*	4.17	7.42	*Bacteroidota*	4.90	5.77
*Bacteroidota*	2.52	3.02	*Patescibacteria*	3.57	5.76
Other	2.88	4.59	*Fusobacteriota*	1.20	2.68
-	-	-	Other	1.77	2.28
Families					
*Moraxellaceae*	57.14	26.85	*Moraxellaceae*	53.92	25.92
*Staphylococcaceae*	7.09	18.82	*Staphylococcaceae*	8.24	17.53
*Cardiobacteriaceae*	5.92	12.55	*Streptococcaceae*	4.65	6.43
*Streptococcaceae*	3.67	6.01	*Cardiobacteriaceae*	3.95	9.60
JGI_0000069-P22_family	3.03	6.77	JGI_0000069-P22_family	2.19	4.26
*Mycoplasmataceae*	2.34	7.76	*Microbacteriaceae*	1.65	2.76
*Microbacteriaceae*	2.23	3.95	*Mycoplasmataceae*	1.50	8.36
Gracilibacteria_family	1.03	3.06	*Corynebacteriaceae*	1.26	4.06
*Micrococcaceae*	1.02	2.44	*Weeksellaceae*	1.20	2.89
Other	16.53	20.39	*Fusobacteriaceae*	1.16	2.67
-	-	-	Gracilibacteria_family	1.15	4.08
-	-	-	Other	19.13	17.57
Genera					
*Moraxella*	56.98	26.89	*Moraxella*	53.80	25.94
*Staphylococcus*	7.01	18.82	*Staphylococcus*	8.14	12.56
*Suttonella*	5.91	12.56	*Streptococcus*	4.65	6.43
*Streptococcus*	3.67	6.01	*Suttonella*	3.93	9.61
JGI_0000069-P22_genus	3.03	6.77	JGI_0000069-P22_genus	2.19	4.26
*Mycoplasma*	2.28	7.76	*Mycoplasma*	1.49	8.36
*Leucobacter*	1.94	3.99	*Leucobacter*	1.34	2.79
Gracilibacteria_genus	1.03	3.06	*Corynebacterium*	1.23	4.06
Other	18.15	21.30	Gracilibacteria_genus	1.15	4.08
-	-	-	*Fusobacterium*	1.15	2.66
-	-	-	*Bergeyella*	1.03	2.75
-	-	-	Other	19.90	18.33

The data represent the mean percentage abundance with standard deviation (SD) across all individuals.

“Other” is the sum of all taxa with ≤ 1% relative abundance at each taxonomic level. Note that the phylum Proteobacteria was recently renamed as Pseudomonadota, Firmicutes as Bacillota and Actinobacteriota as Actinomycetota.

### Richness and alpha diversity estimators

**Figure 1:**
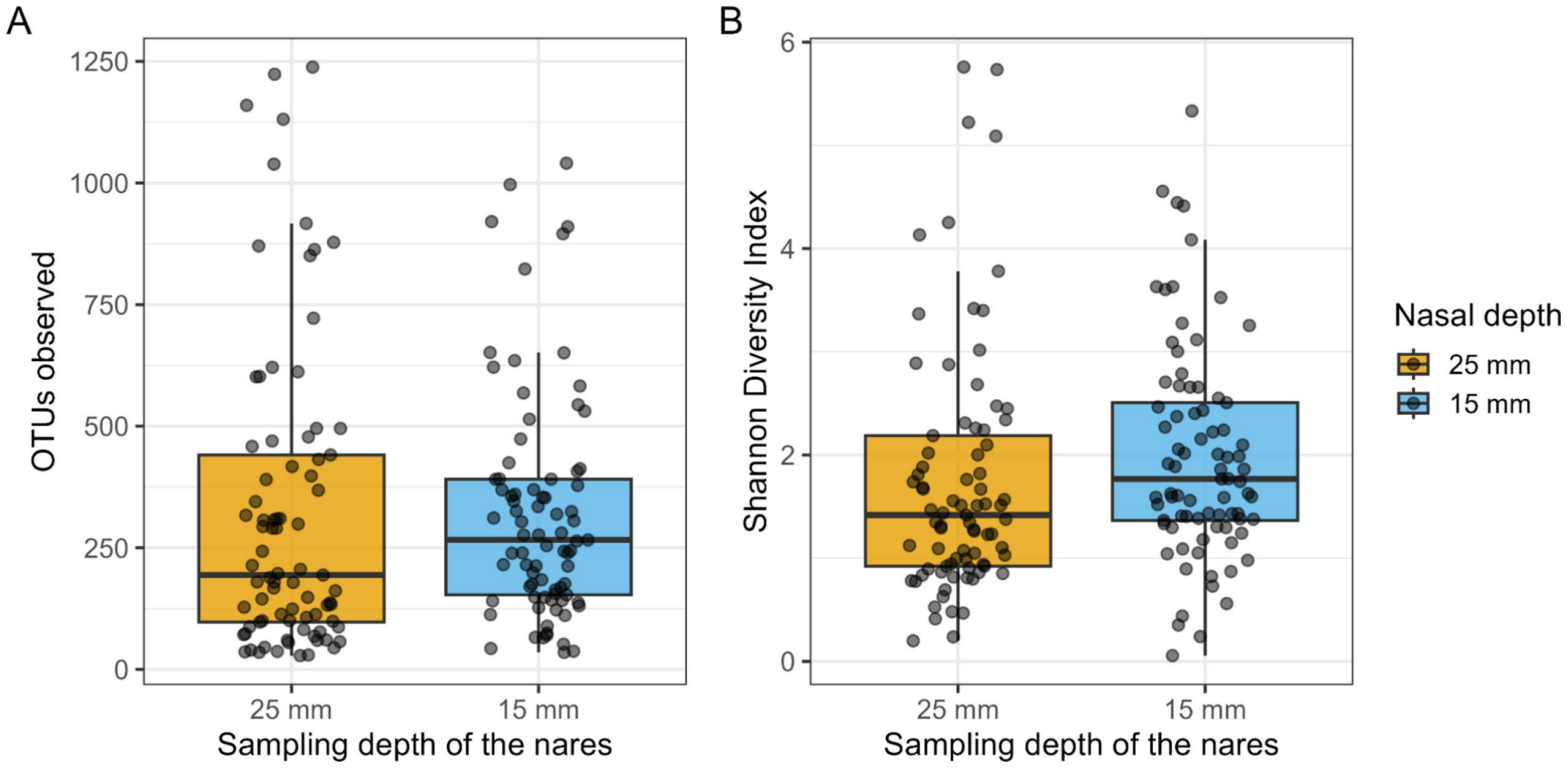
**(A)** The number of nasal bacterial OTUs at the two sampling depths (median, quartiles, minimum/maximum) of healthy awake family dogs. **(B)** The Shannon diversity index of nasal bacterial communities at the two sampling depths (median, quartiles, minimum/maximum) of healthy awake family dogs. Dots represent the individual data points.

There was no significant difference observed in the median number of nasal bacterial OTUs between the two sampling depths (Nasal 25 mm: 194; Nasal 15 mm: 266, median paired difference = 48.8 OTUs; Wilcoxon test *p* = 0.18; see Figure 1A). However, a minor but statistically significant difference was observed in the median Shannon Diversity Index (Nasal 25 mm: 1.42; Nasal 15 mm: 1.77; median paired difference = 0.18; Wilcoxon test *p* = 0.02; see Figure 1B) between the two sampling depths, indicating slightly higher diversity in the outer part of the nose.

### Beta diversity and community composition

**Figure 2:**
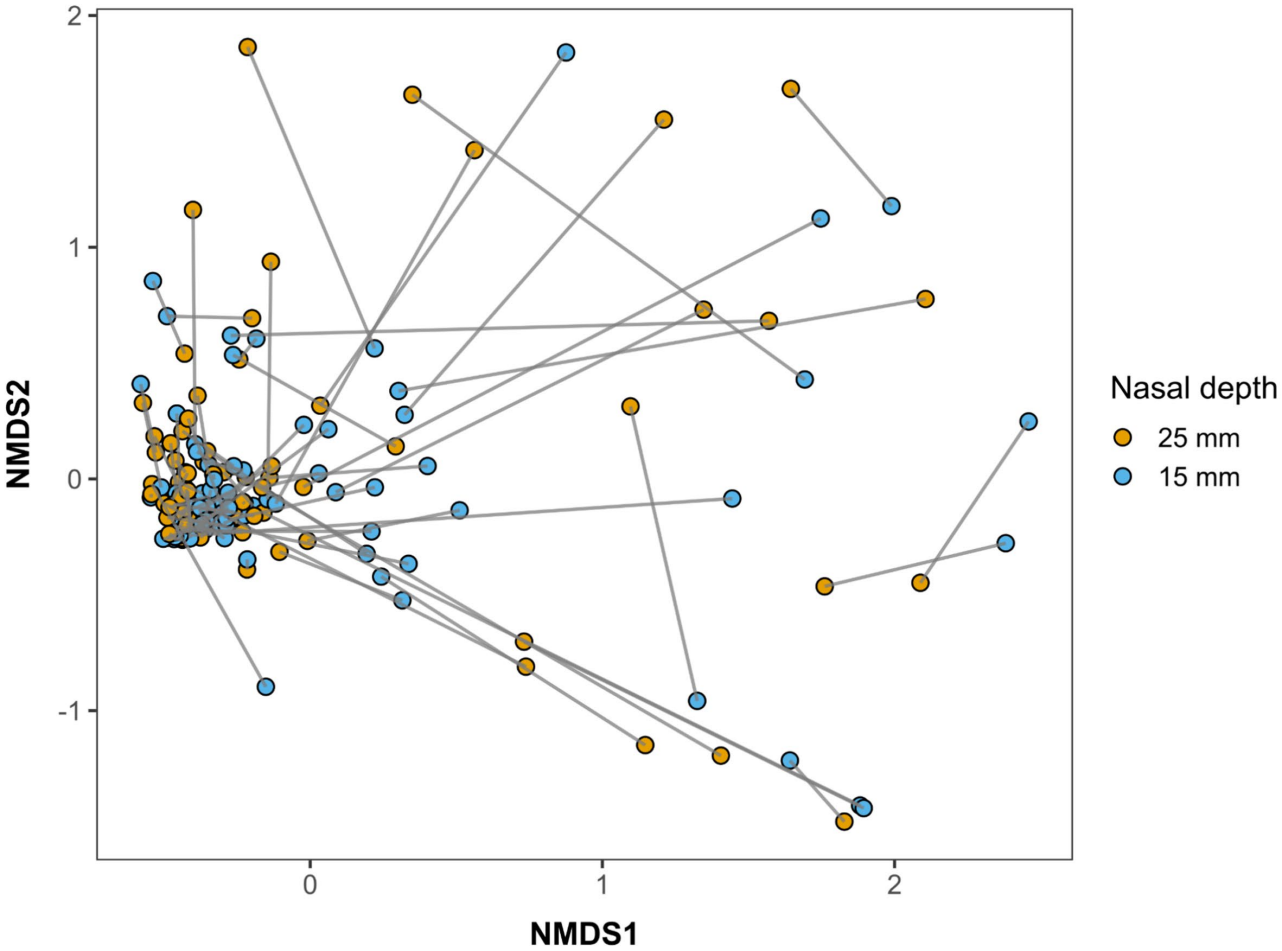
NMDS ordination (stress 0.15) of nasal bacterial OTUs at 25 mm and 15 mm nasal samples from awake family dogs using Bray-Curtis metric. Points represent individual samples, and grey lines connect paired samples from the same dog, highlighting within-individual variation between the two sampling depths.

NMDS revealed no clear separation between the 15 mm and 25 mm nasal samples (stress = 0.15; Figure 2). The ordination showed that bacterial community structure was largely overlapped between the two sampling depths in many individuals, with paired samples from the same dog generally clustering closely in ordination space. This observation was supported by the PERMANOVA results, which indicated no overall significant difference in the bacterial community composition between 15 mm and 25 mm sampling depths (*F* = 1.11, R^2^ = 0.01, *p* = 0.31). However, some individuals showed relatively distinct community composition between the two nasal depths (Figure 2).

Based on the SIMPER analysis, 48% of the dissimilarity between sampling depths was explained by differences in the relative abundance of OTUs affiliated with the genera *Moraxella* (OTU1, 27.2%), *Staphylococcus* (OTU6, 9.6%)*, Suttonella* (OTU7, 7.0%) and *Streptococcus* (OTU8, 4.1%).

## Discussion

Our first aim was to describe the nasal microbiota community composition of a large sample of awake healthy family dogs. The most abundant phylum detected, *Proteobacteria*, matches previous reports (5–8); however, differences were found in the relative abundance. Family *Moraxellaceae* and genus *Moraxella* were identified as major nasal microbiota taxa, similar to other studies (6–8). However, in a study of laboratory beagles, *Mycoplasmataceae* and *Mycoplasma* were found to be equally abundant (5). Despite their relatively high abundance, the role of *Moraxellaceae* and *Moraxella* in the nasal cavity of dogs is still unknown (10).

Previous studies have collected nasal samples from different depths — midway between the tip of the nose and the medial canthus of the eye (5), approximately half an inch (7), or the distal third of the nasal cavity (10). Therefore, our second aim was to compare the nasal microbiota obtained from two different depths (15 mm and 25 mm) of the same dogs. We found no significant differences in bacterial richness based on the number of observed OTUs, but there was a higher Shannon Diversity Index in 15 mm depths. The biological relevance of this difference is unclear, but it can likely be related to the closer proximity to the outside environment. It is known in humans that different parts of the upper airways host transient and resident microorganisms in different ratios due to the different microenvironments created by several factors, like inhaled biological particles, and physical and chemical weathering agents, such as humidity, oxygen, and various immunological or nutritional factors (26). It is possible that the difference we found here in the Shannon Diversity Index is due to the larger number of transient microorganisms (originating from the environment) present in the 15 mm depth. In our study, the sampling order was not randomised: the 25 mm swab was always collected before the 15 mm swab. Thus, one might argue that this may have slightly disturbed the mucosal surface or altered local microbial adherence, potentially increasing similarity between the two samples. This could happen in two ways. First, when taking the first sample (25 mm depth), the swab must pass through the 15 mm region of the nostril, which might inflate the bacterial diversity of that sample because bacteria from both depths may be collected on the swab. Second, when removing the first swab, it again passes through the 15 mm region, so some bacteria sampled at 25 mm depth could be deposited there, potentially increasing the diversity of the second sample. However, the low R^2^ from the PERMANOVA indicates that sampling depth accounted for only < 1% of the total variation in nasal bacterial communities, supporting the conclusion that bacterial composition was largely consistent across the two sampled depths, both exhibiting the same dominant taxa (*Proteobacteria*, *Moraxellaceae*, *Moraxella*). This supports the findings of a human study not detecting a difference in microbial diversity and community composition in three locations (anterior nares, inferior turbinate, and middle meatus; all sampled twice) of the same nostril (27). However, previous dog studies generally collected only one nasal sample per subject, each using slightly different sampling depths, yet reported relatively similar microbial compositions (5–8). Thus, our findings confirm previous results, and we can reasonably assume that the limited depth-related differences observed in our data are unlikely to result from double sampling of the same individuals. But, it is important to note that inter-individual variability could contribute to masking minor or moderate depth-related effects, as nasal microbial communities may be influenced by host-related factors (e.g., disease state, breed characteristics, physiology) and environmental exposures, which can generate variation between individuals (6, 7, 28). Further, according to the NMDS, some individuals appeared to show differences between the two depths, but this difference did not reach significance. This suggests that the overall lack of variation between sampling depths is not primarily due to the method being unable to detect differences in microbiota composition.

These findings suggest that bacterial community composition within the sampled anterior nasal region showed limited detectable differences between 15 mm and 25 mm depths. Nevertheless, it remains possible that both our sampling technique and the sampling methods of earlier studies may have ‘contaminated’ the nasal cavity during sampling, as swabs must pass through the nares to reach deeper regions. Although it is technically easier to collect nasal samples under anaesthesia — when the nostrils can be held open with a speculum and contaminations by the nares and surrounding skin may be avoided (10) — this approach is not feasible for field use. Moreover, sampling under anaesthesia did not yield substantially different results compared to previous studies (6–8), even though previous studies only examined one nasal depth, and none of the studies compared directly the nasal microbiota from shallower depth and a depth with functional relevance. Considering animal welfare aspects during sampling is central to ethical research and veterinary practice. It is important to consider the depth of nasal sampling when swabbing the nares of awake dogs, as deeper insertion may cause discomfort or even pain. Additionally, stress associated with animal handling from longer durations may occur and reduce sample viability, consequently limiting the effective sample size of the study. Thus, less invasive sampling techniques should be prioritized in accordance with international animal welfare standards, such as the EU Directive 2010/63/EU. Given that previous studies reported similar microbial compositions despite varying sampling depths, our results indicate that a shallower sampling depth (around 15 mm) may capture comparable nasal microbiota profiles in field studies, providing representative microbiota profiles while reducing intrusion and discomfort for healthy, awake, mesocephalic dogs.

Our study has several limitations. We acknowledge that a veterinary checkup would be ideal for assessing the health status of the subjects, as owner reports could be less accurate (29). However, the study involved typical family dogs rather than clinical patients, and in such cases, it is common to use and accept the owner’s statements about health, especially in the case of large sample sizes similar to humans (30).

It should be noted that our study focused solely on the effect of different swabbing depths on the detected nasal bacterial communities, and the impact of downstream processes was not tested. We acknowledge that beyond sample collection, DNA extraction methods, choice of PCR primers, the applied bioinformatic pipeline and others (31–34) could have a remarkable influence on the taxa detected. Furthermore, the rapid changes related to various steps of next-generation DNA sequencing protocols [e.g., updated primer sequences, new software and sequencing platforms; Walters et al. (35) and Hu et al. (36)], which are intended to achieve more accurate taxon assignment, higher throughput or improved data quality, are hindering the recommendation of a general complete workflow for the analysis of canine nasal microbiome. Therefore, in this study, we used a sample processing pipeline which is widely used in the analysis of microbial community composition and which was similar to those applied by others for studying the canine nasal microbiome (6–8) and in our previous projects for studying the gut microbiome of dogs (21, 37).

Nevertheless, performing a comprehensive study on the effect of sampling depth and suggesting a less invasive swabbing technique for accurately characterizing healthy canine nasal microbial communities could aid in developing future guidelines for dog microbiome analysis. Moreover, future research may evaluate potential order effects by randomising sampling depth or comparing depths across different individuals. Unfortunately, other current limitations, such as a lack of data and specific references, and the need for expertise to analyse and interpret the results, hinder the direct implementation of the findings in practice. On the other hand, the decreasing cost of DNA sequencing may allow the outcomes of this study to serve as a basis for targeted therapies in the near future. Future studies with larger and more balanced breed samples would be required to evaluate breed-related differences in nasal microbiota more robustly. In the long term, uncovering the possible link between nasal microbiota and olfactory performance, as suggested by others (3, 7), and finding ways to enhance olfactory performance by influencing nasal microbiota community composition could be a goal for practical application.

## Conclusion

In a large sample of family dogs, the nasal microbiota was dominated by the phylum *Proteobacteria*, the family *Moraxellaceae*, and the genus *Moraxella*, consistent with previous (mostly smaller scaled) studies. The Shannon Diversity Index was higher in the 15 mm samples compared to the 25 mm samples indicating closer proximity to the outside environment, but no substantial differences were found in richness or overall microbiota composition between the two sampling depths. Our results indicate that shallow sampling may be a practical option for characterizing nasal microbiota in healthy, awake, mesocephalic dogs, to support both field application and animal welfare. Our results did not detect major differences in richness or overall community composition between 15 mm and 25 mm sampling depths.

## Data Availability

Raw sequence reads have been deposited in the NCBI Sequence Read Archive under BioProject ID PRJNA1371937. All data generated or analysed during this study are included in this published article and in an Excel table named “Supplementary data”.

## References

[1] François A Grebert D Rhimi M Mariadassou M Naudon L Rabot S . Olfactory epithelium changes in germfree mice. Sci Rep. (2016) 6:24687. doi: 10.1038/srep24687, 27089944 PMC4835764

[2] Naudon L François A Mariadassou M Monnoye M Philippe C Bruneau A . First step of odorant detection in the olfactory epithelium and olfactory preferences differ according to the microbiota profile in mice. Behav Brain Res. (2020) 384:112549. doi: 10.1016/j.bbr.2020.112549, 32050097

[3] Jenkins EK DeChant MT Perry EB. When the nose doesn’t know: canine olfactory function associated with health, management, and potential links to microbiota. Front Vet Sci. (2018) 5:56. doi: 10.3389/fvets.2018.00056, 29651421 PMC5884888

[4] Koskinen K Reichert JL Hoier S Schachenreiter J Duller S Moissl-Eichinger C . The nasal microbiome mirrors and potentially shapes olfactory function. Sci Rep. (2018) 8:1296. doi: 10.1038/s41598-018-19438-3, 29358754 PMC5778015

[5] Ericsson AC Personett AR Grobman ME Rindt H Reinero CR. Composition and predicted metabolic capacity of upper and lower airway microbiota of healthy dogs in relation to the fecal microbiota. PLoS One. (2016) 11:e0154646. doi: 10.1371/journal.pone.0154646, 27136381 PMC4852910

[6] Tress B Dorn ES Suchodolski JS Nisar T Ravindran P Weber K . Bacterial microbiome of the nose of healthy dogs and dogs with nasal disease. PLoS One. (2017) 12:e0176736. doi: 10.1371/journal.pone.0176736, 28459886 PMC5411083

[7] Isaiah A Hoffmann AR Kelley R Mundell P Steiner JM Suchodolski JS. Characterization of the nasal and oral microbiota of detection dogs. PLoS One. (2017) 12:e0184899. doi: 10.1371/journal.pone.0184899, 28934260 PMC5608223

[8] Vangrinsven E Fastrès A Taminiau B Billen F Daube G Clercx C. Assessment of the nasal microbiota in dogs with fungal rhinitis before and after cure and in dogs with chronic idiopathic rhinitis. BMC Microbiol. (2023) 23:104. doi: 10.1186/s12866-023-02828-7, 37061685 PMC10105444

[9] Pereira AM Clemente A. Dogs’ microbiome from tip to toe. Top Companion Anim Med. (2021) 45:100584. doi: 10.1016/j.tcam.2021.100584, 34509665

[10] Vangrinsven E Fastrès A Taminiau B Billen F Daube G Clercx C. Variations in facial conformation are associated with differences in nasal microbiota in healthy dogs. BMC Vet Res. (2021) 17:361. doi: 10.1186/s12917-021-03055-w, 34819074 PMC8611846

[11] Bassiouni A Cleland EJ Psaltis AJ Vreugde S Wormald PJ. Sinonasal microbiome sampling: a comparison of techniques. PLoS One. (2015) 10:e0123216. doi: 10.1371/journal.pone.0123216, 25876035 PMC4396979

[12] Georgevsky D Carrasco JJ Valenzuela M McGreevy PD. Domestic dog skull diversity across breeds, breed groupings, and genetic clusters. J Vet Behav. (2014) 9:228–34. doi: 10.1016/j.jveb.2014.04.007

[13] Ferreira SD Almeida GG Silva SD Vogas GP Fujiwara RT De Andrade AS . Nasal, oral and ear swabs for canine visceral leishmaniasis diagnosis: new practical approaches for detection of *leishmania infantum* DNA. PLoS Negl Trop Dis. (2013) 7:e2150. doi: 10.1371/journal.pntd.0002150, 23593518 PMC3617150

[14] Salamon A Baranya E Zsiros LR Miklósi Á Csepregi M Kubinyi E . Success in the natural detection task is influenced by only a few factors generally believed to affect dogs’ olfactory performance. Sci Rep. (2024) 14:12351. doi: 10.1038/s41598-024-62957-5, 38811746 PMC11137087

[15] Salamon A Miklósi Á Zsiros LR Kovács T Kubinyi E Andics A . Breed differences in olfactory performance of dogs. Sci Rep. (2025) 15:2675. doi: 10.1038/s41598-025-87136-y, 39838112 PMC11751464

[16] Salamon A Lakatos B Miklósi Á Csibra B Kubinyi E Andics A . Training level and personality affect border collies’ olfactory performance in the natural detection task. Appl Anim Behav Sci. (2025) 286:106625. doi: 10.1016/j.applanim.2025.106625

[17] Herlemann DP Labrenz M Jürgens K Bertilsson S Waniek JJ Andersson AF. Transitions in bacterial communities along the 2000 km salinity gradient of the Baltic Sea. ISME J. (2011) 5:1571–9. doi: 10.1038/ismej.2011.41, 21472016 PMC3176514

[18] Apprill A McNally S Parsons R Weber L. Minor revision to V4 region SSU rRNA 806R gene primer greatly increases detection of SAR11 bacterioplankton. Aquat Microb Ecol. (2015) 75:129–37. doi: 10.3354/ame01753

[19] Lange-Enyedi NT Borsodi AK Németh P Czuppon G Kovács I Leél-Őssy S . Habitat-related variability in the morphological and taxonomic diversity of microbial communities in two Hungarian epigenic karst caves. FEMS Microbiol Ecol. (2023) 99:161. doi: 10.1093/femsec/fiad161, 38066687

[20] Schloss PD Westcott SL Ryabin T Hall JR Hartmann M Hollister EB . Introducing mothur: open-source, platform-independent, community-supported software for describing and comparing microbial communities. Appl Environ Microbiol. (2009) 75:7537–41. doi: 10.1128/AEM.01541-09, 19801464 PMC2786419

[21] Salamon A Szabó A Felföldi T Bel Rhali S Andics A Miklósi Á . Human-like associations between gut microbiome composition and inattention, hyperactivity, and impulsivity in dogs. BMC Biol. (2025) 23:352. doi: 10.1186/s12915-025-02410-9, 41299418 PMC12661682

[22] R Development Core Team R: A Language and Environment for Statistical Computing (R Foundation for Statistical Computing, Vienna, Austria). (2024). Available online at: https://www.R-project.org/ (Accessed November 10, 2024).

[23] Oksanen J Blanchet FG Friendly M Kindt R Legendre P McGlinn D . Vegan: Community Ecology Package. R Package Version 2.6.10. (2025). Available online at: https://CRAN.R-project.org/package=vegan (Accessed February 22, 2025).

[24] Wickham H. ggplot2: Elegant Graphics for Data Analysis. New York: Springer (2009).

[25] Hammer Ø Harper DAT Ryan PD. Past: paleontological statistics software package for education and data analysis. Palaeontol Electron. (2001) 4:4. Available online at: https://paleo.carleton.ca/2001_1/past/past.pdf

[26] Loperfido A Rizzo D Fionda B Mureddu L Tondo A Tagliaferri L . The potential role of the microbiome in the pathogenesis of nasal Tumors: a comprehensive review. Medicina. (2024) 60:1808. doi: 10.3390/medicina60111808, 39596994 PMC11596812

[27] Biswas K Hoggard M Jain R Taylor MW Douglas RG. The nasal microbiota in health and disease: variation within and between subjects. Front Microbiol. (2015) 9:134. doi: 10.3389/fmicb.2015.00134, 25784909 PMC4345913

[28] Vientós-Plotts AI Ericsson AC Reinero CR. The respiratory microbiota and its impact on health and disease in dogs and cats: a one health perspective. J Vet Intern Med. (2023) 37:1641–55. doi: 10.1111/jvim.16824, 37551852 PMC10473014

[29] Schmid SM Sexton CL Yoerger A Kauffman M McClelland RL Creevy KE . Accuracy of owner-reported diagnoses for dogs enrolled in the dog aging project as compared to veterinary electronic medical records. PLoS One. (2026) 21:e0342427. doi: 10.1371/journal.pone.0342427, 41779687 PMC12959718

[30] Wu S Wang R Zhao Y Ma X Wu M Yan X . The relationship between self-rated health and objective health status: a population-based study. BMC Public Health. (2013) 13:320. doi: 10.1186/1471-2458-13-320, 23570559 PMC3637052

[31] Sipos R Székely AJ Palatinszky M Révész S Márialigeti K Nikolausz M. Effect of primer mismatch, annealing temperature and PCR cycle number on 16S rRNA gene-targetting bacterial community analysis. FEMS Microbiol Ecol. (2007) 60:341–50. doi: 10.1111/j.1574-6941.2007.00283.x, 17343679

[32] Feinstein LM Sul WJ Blackwood CB. Assessment of bias associated with incomplete extraction of microbial DNA from soil. Appl Environ Microbiol. (2009) 75:5428–33. doi: 10.1128/AEM.00120-09, 19561189 PMC2725469

[33] Klindworth A Pruesse E Schweer T Peplies J Quast C Horn M . Evaluation of general 16S ribosomal RNA gene PCR primers for classical and next-generation sequencing-based diversity studies. Nucl Acids Res. (2013) 41:e1. doi: 10.1093/nar/gks808, 22933715 PMC3592464

[34] Fares M Tharwat EK Cleenwerck I Monsieurs P Van Houdt R Vandamme P . The unresolved struggle of 16S rRNA amplicon sequencing: a benchmarking analysis of clustering and denoising methods. Environ Microbiome. (2025) 20:51. doi: 10.1186/s40793-025-00705-6, 40361240 PMC12076876

[35] Walters W Hyde ER Berg-Lyons D Ackermann G Humphrey G Parada A . Improved bacterial 16S rRNA gene (V4 and V4-5) and fungal internal transcribed spacer marker gene primers for microbial community surveys. mSystems. (2016) 1:10–128. doi: 10.1128/msystems.00009-15, 27822518 PMC5069754

[36] Hu T Chitnis N Monos D Dinh A. Next-generation sequencing technologies: an overview. Hum Immunol. (2021) 82:801–11. doi: 10.1016/j.humimm.2021.02.012, 33745759

[37] Kubinyi E Bel Rhali S Sándor S Szabó A Felföldi T. Gut microbiome composition is associated with age and memory performance in pet dogs. Animals. (2020) 10:1488. doi: 10.3390/ani10091488, 32846928 PMC7552338

