# Supplementary material for: Nasal microbiota profiles are similar at two swabbing depths in healthy awake dogs
Source: Front Vet Sci. 2026 Apr 28;13:1795324. doi: 10.3389/fvets.2026.1795324 (PMC13160798; doi:10.3389/fvets.2026.1795324)
Supplement: Supplementary file 2 [file Table_1.docx]

**Supplementary information to:**

**Nasal microbiota profiles are similar at two swabbing depths in healthy awake dogs**

*Correspondence: dr.attila.salamon@gmail.com, salamon.attila@ttk.elte.hu

**Supplementary Table 1.** Information about controls and procedures applied during the study.

Categories are based on Fierer et al. (2025).

| **Area** | **Term** | **Included in this study** |
| --- | --- | --- |
| Sample collection | Sampling controls | No |
|  | Extraction controls | No |
|  | Library preparation controls | Yes: blanks |
| Laboratory methods | Workspace decontamination | Yes: cleaning with water and 70% ethanol, laminal flow hood, UV-treatment |
|  | Personal protective equipment | Yes: lab coat, gloves, eye protection |
|  | Positive controls | Yes: PCR amplification controls, re-sequenced samples |
| Data processing | Decontamination pipeline | Yes:  The mothur v1.48 tool was utilized using the MiSeq SOP (http://www.mothur.org/wiki/MiSeq_SOP) downloaded on 8th January 2024 as referred in Salamon et al. (2025). We carried out the following steps removing low quality and ambiguous sequences:  - The deltaq parameter of the ‘make.contigs’ command was adjusted to 10 for additional quality filtering to eliminate sequencing errors. Reads containing ambiguous base calls (Ns) was also filtered out from the sequence set  - Primers were removed from the start and the end of the sequences, and singletons were also removed from the dataset, according to Kunin et al. (2010).  - Chimeras were identified and removed using the mothur-implemented version of VSEARCH.  - Denoising was performed using mothur’s pre.cluster command using the default algorithm applying the suggested 4-bp difference cutoff.  - Taxonomic assignment was carried out applying 1000 iterations and a minimum bootstrap confidence score of 80%.  - Reads of non-bacterial origin (e.g. Archaea, chloroplasts, mitochondria, Eukaryota, unknown) were identified as contaminants and removed from the dataset. |
|  | Contaminant database | For sequence alignment and taxonomic assignments, the ARB-SILVA SSU Ref NR 138 reference database (Quast et al. 2013) was used. The same database was used for reference for contaminating lineages. |
| Datasets | Control sequences | No |
|  | Original dataset | Raw sequence reads have been deposited in the NCBI Sequence Read Archive under BioProject ID PRJNA1371937. |
|  | Processed dataset | Not provided |

**References**

Fierer N, Leung PM, Lappan R, Eisenhofer R, Ricci F, Holland SI, et al. Guidelines for preventing and reporting contamination in low-biomass microbiome studies. *Nature Microbiol*. (2025) 10:1570-80. doi: 10.1038/s41564-025-02035-2

Kunin V, Engelbrektson A, Ochman H, Hugenholtz P. Wrinkles in the rare biosphere: pyrosequencing errors can lead to artificial inflation of diversity estimates. *Environ Microbiol.* (2010) 12:118-123. doi: 10.1111/j.1462-2920.2009.02051.x

Quast C, Pruesse E, Yilmaz P, Gerken J, Schweer T, Yarza P, et al. The SILVA ribosomal RNA gene database project: improved data processing and web-based tools. *Nucl Acids Res.* (2013) 41:D590-D596.

Salamon, A, Szabó, A, Felföldi, T, Bel Rhali, S, Andics, A, Miklósi, Á, et al. Human-like associations between gut microbiome composition and inattention, hyperactivity, and impulsivity in dogs. *BMC Biol*. (2025) 23:352. doi: 10.1186/s12915-025-02410-9
